# Significance of miR-141 and miR-340 in cervical squamous cell carcinoma

**DOI:** 10.1515/med-2021-0281

**Published:** 2021-06-16

**Authors:** Wenting Li, Bo Yang, Yiqun Li, Cuicui Wang, Xinzhi Fang

**Affiliations:** Department of Pathology, Shenzhen University General Hospital, No. 1098 Xueyuan Road, Nanshan District, Shenzhen 518055, China; Department of Pathology, Affiliated Tumor Hospital of Xinjiang Medical University, Urumqi 830011, Xinjiang, China

**Keywords:** cervical squamous cell carcinoma, high-grade squamous intraepithelial lesion, miR-141, miR-340, PTEN

## Abstract

**Background:**

We investigated the expression and clinical significance of miR-141 and miR-340 in cervical squamous cell carcinoma (CSCC).

**Methods:**

Expression of miR-141 and miR-340 in CSCC, high-grade squamous intraepithelial lesion (HSIL), and normal cervical squamous epithelium were detected by qRT-PCR. PTEN was assessed by immunohistochemistry. Their relationship with clinicopathological features was analyzed.

**Results:**

The changes of miR-141 and miR-340 were different in CSCC, HSIL, and normal squamous epithelium (*P* = 0.030). miR-141 expression was statistically significant in gross type, differentiation, uterine corpus invasion, nerve invasion, vagina invasion, and FIGO stage in CSCC (*P* < 0.05). miR-340 expression was related to tumor size, differentiation, nerve invasion, lymph node metastasis, and FIGO stage in CSCC (*P* < 0.05). miR-141 and miR-340 expressions were statistically significant in different ages (*P* < 0.05) in HSIL. The AUC of miR-141 in CSCC diagnosis and that of miR-340 in HSIL diagnosis were 0.893 and 0.764, respectively. The sensitivity and the specificity of miR-141 for diagnosis of CSCC were 95.0% and 60.8%, respectively, while those of miR-340 for diagnosis of HSIL were 90.0 and 48.6%, respectively. miR-141 and miR-340 expressions are associated with PTEN expression (*P* = 0.002 and *P* < 0.001).

**Conclusion:**

miR-141 and miR-340 may be associated with their target gene PTEN and involved in the carcinogenesis of cervical squamous epithelium.

## 1 Introduction

Cervical cancer is one of the most common malignancies in women. The latest statistics show there are more than half a million new cases and more than 300,000 deaths every year worldwide and about 100,000 new cases of cervical cancer and 30,000 deaths in China [1]. According to the 2018 data, the global incidence of cervical cancer is 13 per 100,000 people. Among the histological types of cervical cancer, cervical squamous cell carcinoma (CSCC) accounts for about 90% [2].

The most involved molecular mechanisms of CSCC development include mutations in tumor suppressor genes (such as p53, p16, and PTEN), genetic susceptibility, chromosomal translocation, and single nucleotide polymorphism [3]. The study of microRNA (miRNA) in the occurrence of malignant tumors has opened up new directions for elucidating the molecular mechanism of the cancerization from cervical epithelial lesions to CSCC. miRNA is a type of highly conserved noncoding small RNA (18–25 nucleotides) that can bind to the 3′ untranslated region of the corresponding mRNA, thus inhibiting translation or promoting degradation of the corresponding mRNA and silencing gene expression after transcription [4]. miRNAs have various types, and they are involved in the regulation of nearly one-third of protein-coding genes. They also have the characteristics of multiple targets and tissue specificity. miRNA can regulate multiple target genes, and one target gene can be regulated by multiple miRNAs. Currently, it is believed that miRNA regulates a variety of tumor-related genes and participates in tumorigenesis through regulating oncogenes and tumor suppressor genes [5]. Some miRNAs play a role similar to tumor suppressor genes, such as miR-125, miR-15a, miR-143, miR-145, and miR-340, while some function as oncogenes, such as miR-21, miR-17, miR-18a, miR141, miR-155, and miR-19a.

The tumor suppressor gene PTEN/MMAC1/TEP1 (phosphatase and tensin homolog deleted on chromosome Ten/mutated in multiple advanced cancer/TGF beta regulated and epithelial cell-enriched phosphatase1) is located on human chromosome 10q23.3, with a total length of 200 kb. It has nine exosomes and eight introns. It is the first identified tumor suppressor gene with the dual specific phosphatase activity, and its structural and functional abnormalities are commonly found in many human tumors [6]. Our previous study [7] found that the PTEN gene was a predictive target gene for multiple miRNAs in endometrial cancer. We wonder if PTEN plays the same role in cervical cancer.

In this study, we first detected miR-141 and miR-340 expressions in CSCC by RT-PCR as well as PTEN expression by immunohistochemistry and then analyzed their relationship with clinicopathological parameters. Furthermore, we explored the mechanism and significance of miR-141 and miR-340 in the transformation process from cervical precancerous lesions (intraepithelial lesions) to cancerous malignancy. Our findings may provide important clues for exploring the key molecules of squamous intraepithelial lesion progression to CSCC and may lay the foundation for the risk prediction of CSCC, as well as the study of the prognosis, and cancerization of cervical intraepithelial lesions.

## 2 Materials and methods

### 2.1 Tissues

In total, 104 patients with CSCC and 20 patients with high-grade squamous intraepithelial lesions (HSILs) who were admitted to Affiliated Tumor Hospital of Xinjiang Medical University were enrolled in this study. Surgical treatment was the primary therapeutic option in all cases. The cancer tissue specimens (*n* = 104) were obtained by surgical resection. The inclusion criteria were as follows: (1) patients were with CSCC or HSILs of the first onset diagnosed with cytology and/or histological biopsy and/or postoperative pathology. (2) Patients did not receive treatment before surgery, including chemotherapy, radiotherapy, and endocrine therapy. (3) Patients had no history of other malignant tumors or genetic diseases. (4) Patients were with qualified tissue specimens that were processed by standard specifications. The exclusion criteria were as follows: (1) Patients with an unclear diagnosis of CSCC or HSILs. (2) Patients with histological tumor types other than CSCC and HSILs. (3) Patients who had received radiotherapy, chemotherapy, and targeted drug therapy before surgery. (4) Patients with the history of other malignant tumors and genetic diseases. (5) Patients with unqualified tissue specimens. For control, ten subjects who underwent total hysterectomy because of uterine fibroids were enrolled. Normal cervical squamous tissues were collected from these ten control subjects. One hundred and four CSCC tissue samples were used for the detection of PTEN expression. Genomic DNA was extracted from tissue samples of 20 CSCCs, 20 high-grade squamous intraepithelial lesions (HSILs), and 10 normal cervical squamous tissues (controls). The clinical data of ethnic group, age, lymph node metastases, grade of cervical carcinomas, FIGO stage, histopathological type, gross type, tumor size, differentiation, invasion depth, myometrial invasion, uterine corpus invasion, vascular invasion, nerve invasion, and vagina invasion were collected (Table 1). The 2009 International Federation of Gynecology and Obstetrics (FIGO) guidelines and the 2014 World Health Organization criteria [2] were used for the classification of clinical staging and histopathological type of cervical carcinoma. The local Ethics Committee approved this study.

**Table 1:** Clinicopathological characteristics of subjects included in the study

Clinicopathological features		CSCC (*n* = 104)	HSILs (*n* = 20)	Control (*n* = 10)
Age (year)	≤50	71	12	5
	>50	33	8	5
Ethnic group	Han	57	10	5
	Uygur	47	10	5
Gross type	Exogenous/nipples	36	NA	NA
	Endogenous/infiltrated	68	NA	NA
Tumor size	≤4 cm	87	NA	NA
	>4 cm	17	NA	NA
Differentiation	Low grade	62	NA	NA
	High grade	42	NA	NA
Invasion depth	Less than full thickness	64	NA	NA
	Reach full thickness	40	NA	NA
Uterine corpus invasion	Non-invasive	85	NA	NA
	Invasive	19	NA	NA
Vascular tumor thrombus	No tumor thrombus	61	NA	NA
	Have tumor thrombus	43	NA	NA
Nerve invasion	Non-invasive	96	NA	NA
	Invasive	8	NA	NA
Vagina invasion	Non-invasive	99	NA	NA
	Invasive	5	NA	NA
Lymph node metastasis	Non-metastasis	81	NA	NA
	Metastasis	23	NA	NA
FIGO stage	Stage I	76	NA	NA
	Stage II	28	NA	NA

Note: CSCC, cervical squamous cell carcinoma; HSILs, high-grade squamous intraepithelial lesions; NA, not applicable.

### 2.2 qRT-PCR

Total RNA was extracted using the RNeasy FFPE kit (Qiagen, Beijing, China). The Nanodrop-2000 spectrophotometer (UV-2800H, UNICO, USA) was used to determine the RNA quality and concentration. Next, RNA was reverse transcribed into cDNA. The reaction system was (15 μL in total): 10 ng RNA sample (5 μL × 2 ng/mL), 3 μL reverse transcription primer, 1.5 μL 10 × RT buffer, 0.15 μL dNTPs (100 mmol/L), 1 μL Muhiscribe^TM^ reverse transcriptase, 0.19 μL 20 U/μL RNase inhibitor, and 4.16 μL ribozyme-free water. The reaction conditions were as follows: 16°C for 30 min, 42°C for 30 min, and 85°C for 5 min. The single-tube TaqMan miRNA assays were used to detect and quantify mature miRNAs. U6 small nuclear RNA (Ambion, Austin, TX, USA) was used as an internal normalization control. The relative quantity of each miRNA was calculated by the comparative CT (2^−ΔΔCt^) method, in which ΔΔCt was calculated as follows:

\text{ΔΔCt}=(\text{Ct}\hspace{.5em}\text{miR-of-interest}\mbox{--}\text{Ct}\hspace{.5em}\text{U}6)\hspace{.5em}\text{cancer}\mbox{--}(\text{Ct}\hspace{.5em}\text{miR-of-interest}\mbox{--}\text{Ct}\hspace{.5em}\text{U}6)\hspace{.5em}\text{control}\text{.}]

### 2.3 Immunohistochemical staining

Immunohistochemistry was performed using the PV-6000 kit (Beijing Zhong Shan-Golden Bridge Biological Technology CO., Ltd, Beijing, China) according to the instructions. The antibody was anti-PTEN (Cat# 138G6, 1:500, Cell Signaling, USA). The color development was performed with DAB. After counterstaining with hematoxylin, the sections were observed under the microscope. The relative PTEN expression level was presented as the immunoreactive score, which was evaluated according to the positive staining percentage and staining intensity. The positive staining percentage was defined as: 0–1%, 0 point; 1–10%, 1 point; 11–33%, 2 points; 33–66%, 3 points; and ≥66% positive cells, 4 points. The staining intensity was evaluated as follows: negative staining, 0 point; weak staining, 1 point; moderate staining, 2 points; strong staining, 3 points. The immunoreactive score was 0–12. When the immunoreactive score was ≤3, it was considered as PTEN loss [8]. The ones without incubation of primary antibodies were used as negative controls.

### 2.4 Statistical analysis

SPSS (version 17.0; SPSS Inc., IL, USA) was used for statistical analyses. Differences between the variables were statistically evaluated using the Student’s *t*-test and Chi-square test. *P* (two tailed) <0.05 indicates a statistically significant difference.

## 3 Results

### 3.1 Expression of miR-141 and miR-340 in cervical cancer

miR-141 expression was mostly upregulated in CSCC. It showed a gradual downward trend in miR-141 expression in CSCC (4.76 ± 0.37), HSIL (−0.15 ± 0.71), and normal squamous epithelium (−0.26 ± 0.49). Statistical analysis showed that miR-141 expression between CSCC and normal squamous epithelial tissue was statistically significant (*P* = 0.035). At the same time, the difference in expression of miR-141 between HSIL and normal squamous epithelial tissues was also statistically significant (*P* = 0.008). However, miR-141 expression between HSIL and CSCC was not statistically significant (*P* = 0.378) (Table 2).

**Table 2:** miR-141 in CSCC, HSIL, and normal squamous epithelium

miR-141	log_2_ relative quantity (mean ± SE)	*t* value	*P*
Normal	−0.26 ± 0.49		
HSIL	−0.15 ± 0.71	8.020	0.008
CSCC	4.76 ± 0.37	4.361	0.035
CSCC compared with HSIL, *t* = 0.787, *P* = 0.378			

Note: CSCC, cervical squamous cell carcinoma; HSIL, high-grade intraepithelial lesions.

Unlike miR-141, miR-340 expression was mostly downregulated in CSCC. miR-340 expression showed a gradual upward trend in CSCC (−1.48 ± 0.23), HSIL (1.00 ± 0.38), and normal squamous epithelium (3.58 ± 0.99). Statistical analysis showed that miR-340 expression in CSCC was significantly lower than that in the normal squamous cell epithelial tissue (*P* = 0.014). Meanwhile, the difference in miR-340 expression between HSIL and normal squamous epithelial tissue was also statistically significant (*P* = 0.041). However, no statistically significant difference in miR-340 expression was found between HSIL and CSCC (*P* = 0.860; Table 3).

**Table 3:** miR-340 in cervical squamous cell carcinoma, high-grade intraepithelial lesions, and normal squamous epithelium

miR-340	log_2_ relative quantity (mean ± SE)	*t* value	*P*
Normal	3.58 ± 0.99		
HSIL	1.00 ± 0.38	4.567	0.041
CSCC	−1.48 ± 0.23	6.398	0.014
CSCC compared with HSIL, *t* = 0.031, *P* = 0.860			

Note: CSCC, cervical squamous cell carcinoma; HSIL, high-grade intraepithelial lesions.

### 3.2 Relationship between miR-141/miR-340 expression and clinicopathological features of CSCC patients

In CSCC, miR-141 expression was closely related to gross type (*P* = 0.039), differentiation (*P* = 0.037), uterine corpus invasion (*P* = 0.001), nerve invasion (*P* = 0.010), vagina invasion (*P* = 0.038), and FIGO stage (*P* < 0.001) (Table 4). However, although miR-141 had a certain correlation with lymph node metastasis, there was no significant difference (*P* = 0.063). In addition, miR-141 was not significantly related to age, ethnic group, tumor size, invasion depth, and vascular invasion (all *P* > 0.05).

**Table 4:** Relationship between miR-141/miR-340 and clinicopathological features of CSCC

CSCC clinicopathological features		log_2_ relative quantity of miR-141 (mean ± SE)	log_2_ relative quantity of miR-340 (mean ± SE)
Age	≤50	4.79 ± 0.47	−1.80 ± 0.27
	>50	4.69 ± 0.58	−0.72 ± 0.39
	*t*	0.876	0.031
	*P*	0.353	0.861
Ethnic group	Han	3.17 ± 0.43	−1.27 ± 0.32
	Uygur	6.35 ± 0.44	−1.68 ± 0.34
	*t*	0.167	0.336
	*P*	0.684	0.565
Gross type	Exogenous and papillary	4.01 ± 0.45	−2.03 ± 0.45
	Endogenous and invasion	5.16 ± 0.50	−1.17 ± 0.26
	*t*	4.456	1.841
	*P*	0.039	0.180
Tumor size	≤2.5 cm	5.19 ± 0.39	−1.45 ± 0.24
	>2.5 cm	3.46 ± 0.81	−1.56 ± 0.60
	*t*	2.424	6.082
	*P*	0.125	0.017
Differentiation	Low grade	4.66 ± 0.42	−1.18 ± 0.35
	High grade	4.88 ± 0.64	−1.84 ± 0.29
	*t*	4.539	8.367
	*P*	0.037	0.005
Invasion depth	Not reaching full thickness	5.59 ± 0.55	−2.23 ± 0.32
	Reaching full thickness	3.93 ± 0.45	−0.72 ± 0.28
	*t*	2.745	0.008
	*P*	0.103	0.930
Uterine corpus invasion	No	4.97 ± 0.42	−1.35 ± 0.24
	Yes	3.58 ± 0.18	−2.21 ± 0.75
	*t*	12.818	2.552
	*P*	0.001	0.116
Vascular invasion	No	4.70 ± 0.47	−1.88 ± 0.29
	Yes	4.86 ± 0.59	−0.73 ± 0.34
	*t*	0.014	0.054
	*P*	0.905	0.817
Nerve invasion	No	4.89 ± 0.40	−1.44 ± 0.23
	Yes	3.56 ± 0.28	−1.75 ± 1.10
	*t*	7.005	8.061
	*P*	0.010	0.006
Vagina invasion	No	4.83 ± 0.40	−1.57 ± 0.24
	Yes	4.11 ± 0.46	−0.64 ± 0.77
	*t*	4.552	0.299
	*P*	0.038	0.587
Lymph node metastasis	No	4.81 ± 0.44	−1.61 ± 0.23
	Yes	4.52 ± 0.49	−0.95 ± 0.71
	*t*	3.588	12.027
	*P*	0.063	0.001
FIGO stage	I–II stage	5.08 ± 0.48	−1.44 ± 0.25
	III–IV stage	3.80 ± 0.22	−1.58 ± 0.57
	*t*	18.076	5.186
	*P*	0.000	0.026

Note: CSCC, cervical squamous cell carcinoma.

miR-340 expression was significantly related to the gross size (*P* = 0.017), differentiation (*P* = 0.005), nerve invasion (*P* = 0.006), lymph node metastasis (*P* = 0.001), and FIGO stage (*P* = 0.026; Table 4). However, there were no statistically significant difference in groups of age, ethnic group, gross type, invasion depth, uterine corpus invasion, vascular invasion, and vagina invasion (all *P* > 0.05).

### 3.3 Correlation and significance of miR-141/miR-340 expression in the diagnosis of CSCC and HSIL

Pearson correlation analysis showed that miR-141 and miR-340 expression levels were negatively correlated in CSCC (*R* = −0.480), but the difference was not significant (*P* = 0.092). In addition, in HSIL, miR-141 and miR-340 expression levels were also negatively correlated (*R* = −0.466); however, the difference was also not statistically significant (*P* = 0.178; Table 5).

**Table 5:** Correlation of miR-141/miR-340 in CSCC and HSIL

		miR-340, R(*P)*
CSCC	miR-141	−0.480(0.092)
HSIL	miR-141	−0.446(0.178)

Note: CSCC, cervical squamous cell carcinoma; HSIL, high-grade intraepithelial lesions.

**Figure 1:**
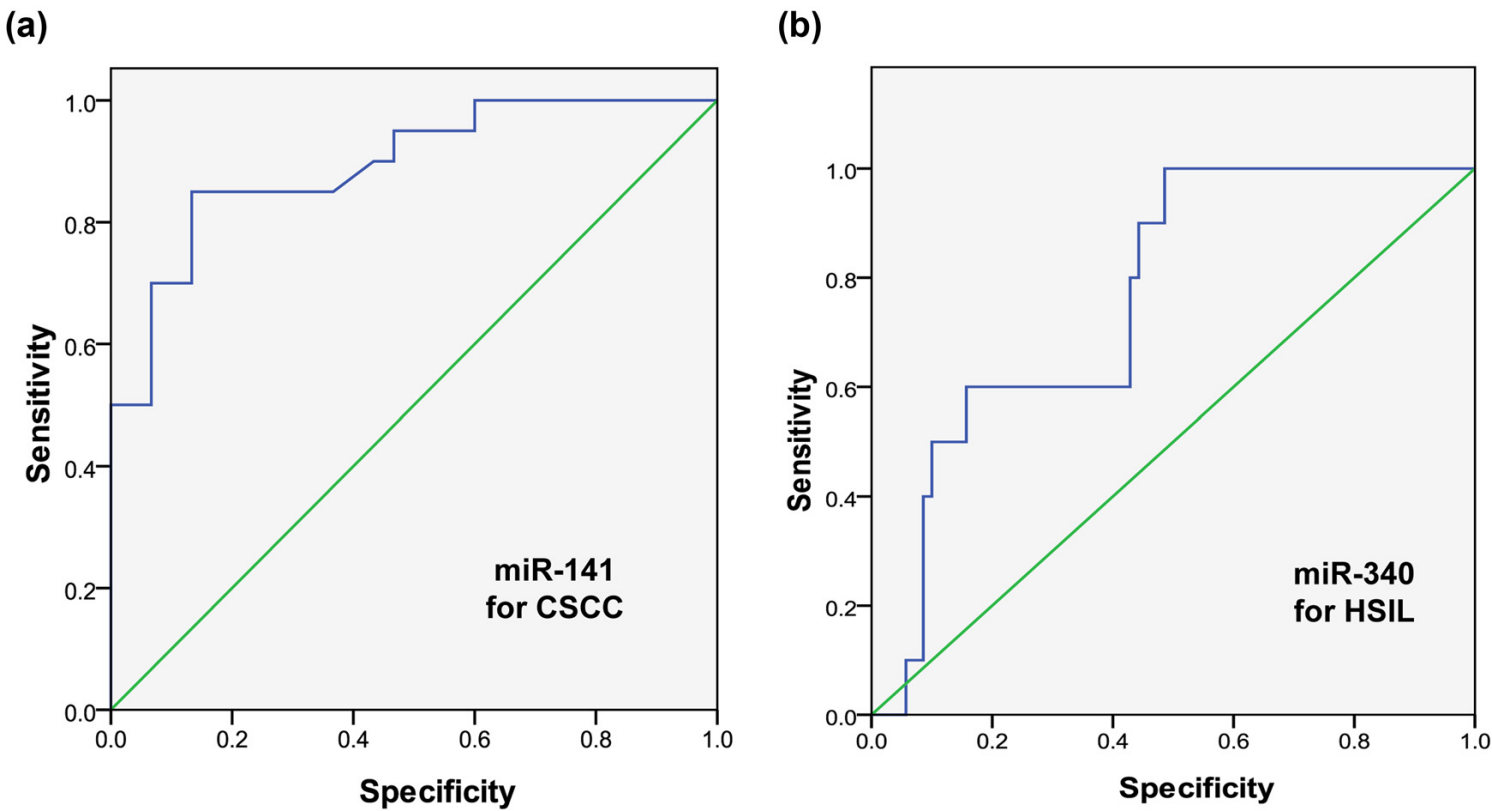
ROC curves of miR-141 for CSCC diagnosis (a) and miR-340 for HSIL diagnosis (b).

At the optimal cut-off point, the sensitivity for the diagnosis of CSCC with miR-141 was 95.0%, and the specificity was 60.8%. The area under the curve was 0.893 (Figure 1a). However, the sensitivity of miR-340 for the diagnosis of HSIL was 90.0%, and the specificity was 48.6%; and the area under the curve was 0.764 (Figure 1b; Table 6).

**Table 6:** The significance of miR-141 in CSCC and miR-340 in HSIL

miRNA	Diagnosis	Area under curve	Standard error	*P*	95% CI	
					Lower limit	Upper limit
miR-141	CSCC	0.893	0.033	0.000	0.828	0.959
miR-340	HSIL	0.764	0.053	0.000	0.660	0.869

Note: CSCC, cervical squamous cell carcinoma; HSIL, high-grade intraepithelial lesions.

### 3.4 PTEN expression in CSCC

**Figure 2:**
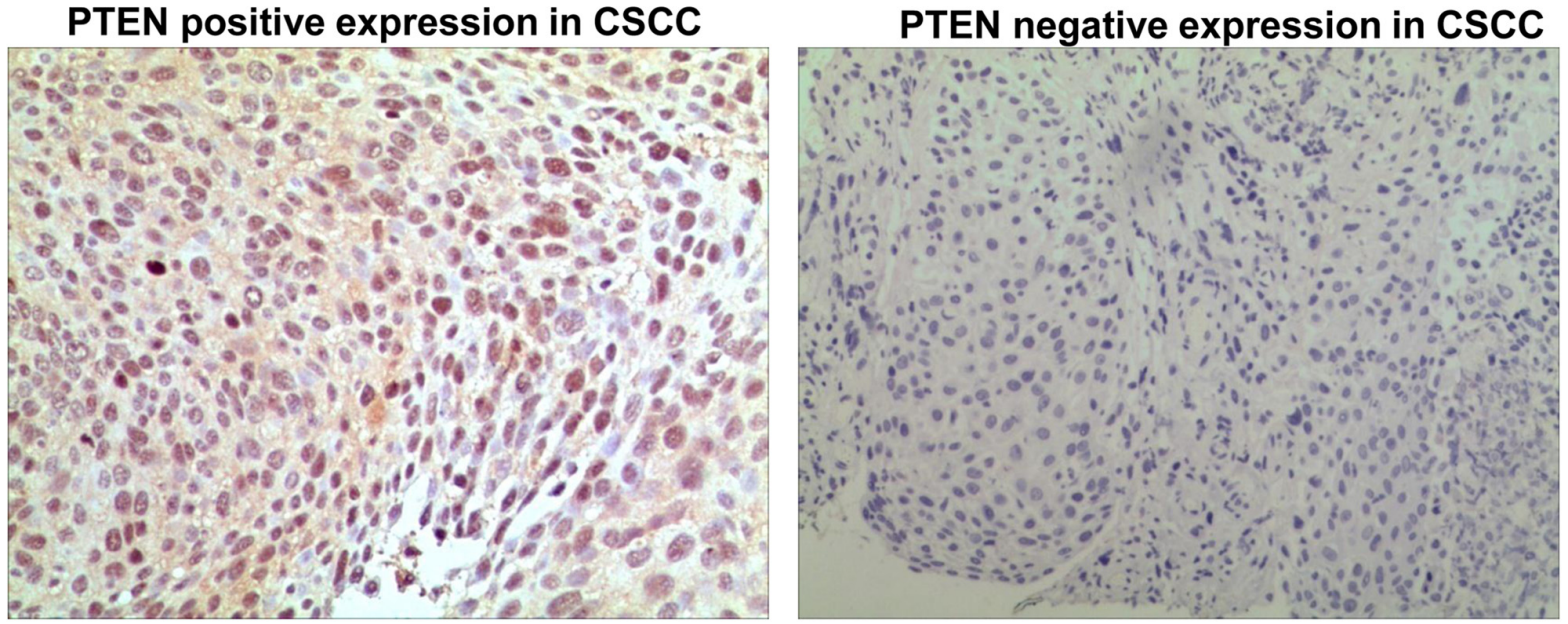
PTEN expression in CSCC. Magnification: ×10.

As the target gene of miR-141 and miR-340, PTEN was expressed in the cytoplasm and the nucleus of CSCC (Figure 2). The positive rate of PTEN in CSCC was 42.3% (44/104).

### 3.5 Relationship between PTEN expression and clinicopathological features of CSCC

In 104 cases of CSCC tissues, we found that PTEN expression was different in different age groups. However, there was no significant difference (*P* = 0.083). Moreover, there was no significant correlation between PTEN and other clinicopathological parameters, including ethnic group (*P* = 0.464), gross type (*P* = 0.691), tumor size (*P* = 0.996), differentiation (*P* = 0.752), invasion depth (*P* = 0.641), uterine corpus invasion (*P* = 0.628), vascular invasion (*P* = 0.136), nerve invasion (*P* = 0.865), vagina invasion (*P* = 0.554), lymph node metastasis (*P* = 0.582), and FIGO stage (*P* = 0.597; Table 7).

**Table 7:** Relationship between PTEN and clinicopathological features of CSCC

		PTEN	
CSCC clinicopathological features		Loss of expression	Positive
Age	≤50	34	38
	>50	21	11
	*χ* ^2^	3.011	
	*P*	0.083	
Ethnic group	Han	32	25
	Uygur	23	24
	*χ* ^2^	0.537	
	*P*	0.464	
Gross type	Exogenous and papillary	20	16
	Endogenous and invasion	35	33
	*χ* ^2^	0.158	
	*P*	0.691	
Tumor size	≤4 cm	46	41
	>4 cm	9	8
	*χ* ^2^	0.000	
	*P*	0.996	
Differentiation	Low grade	32	30
	High grade	23	19
	*χ* ^2^	0.100	
	*P*	0.752	
Invasion depth	Not reaching full thickness	35	29
	Reaching full thickness	20	20
	*χ* ^2^	0.217	
	*P*	0.641	
Uterine corpus invasion	No	44	41
	Yes	11	8
	*χ* ^2^	0.234	
	*P*	0.628	
Vascular invasion	No	36	25
	Yes	19	24
	*χ* ^2^	2.226	
	*P*	0.136	
Nerve invasion	No	51	45
	Yes	4	4
	*χ* ^2^	0.029	
	*P*	0.865	
Vagina invasion	No	53	46
	Yes	2	3
	*χ* ^2^	0.350	
	*P*	0.554	
Lymph node metastasis	No	44	37
	Yes	11	12
	*χ* ^2^	0.303	
	*P*	0.582	
FIGO stage	I–II stage	39	37
	III–IV stage	16	12
	*χ* ^2^	0.279	
	*P*	0.597	

Note: CSCC, cervical squamous cell carcinoma.

### 3.6 Relationship between PTEN and miR-141/miR-340 in CSCC

In CSCC, the expression of miR-141 between patients with loss of PTEN expression (4.19 ± 0.62) and those with PTEN positive expression (5.33 ± 0.37) was statistically significant (*P* = 0.002). The expression of miR-340 between patients with loss of PTEN expression (−1.50 ± 0.23) and those with PTEN positive expression (−1.45 ± 0.41) was also statistically significant (*P* < 0.001; Table 8).

**Table 8:** Relationship between PTEN and miR-141/miR-340 levels in CSCC

		miR-141 log_2_ relative quantity (mean ± SE)	miR-340 log_2_ relative quantity (mean ± SE)
PTEN	Loss of expression	4.19 ± 0.62	−1.50 ± 0.23
	Positive	5.33 ± 0.37	−1.45 ± 0.41
	*t*	10.249	26.595
	*P*	0.002	<0.001

## 4 Discussion

Cervical cancer is one of the most common female malignant tumors in developing countries and is also one of the main causes of deaths in females [9]. Currently, the pathogenesis of CSCC has been extensively studied. However, the role of miRNA in the occurrence of malignant tumors has opened up new directions for elucidating the molecular mechanism of cervical cancer. Moreover, various abnormalities of miRNAs have been found in cervical cancer [10].

MicroRNA are small, single-stranded, noncoding RNAs that can act as oncogenes or tumor suppressor genes in the progression of tumors. Studies have shown that miRNA is differentially expressed in cervical cancer tissue, cervical intraepithelial neoplasia, and normal cervical tissue [11]. Moreover, miRNA is related to the occurrence, metastasis, and invasion of cervical cancer, which may be used as markers for the treatment and prognosis. Thus, miRNAs provide potential new targets for targeted tumor therapy. It is found that the relative expression of miR-141 mRNA in bladder cancer tissue was significantly higher than that in normal tissue adjacent to cancer [12]. However, the expression levels of miR-141 and miR-340 as well as their target gene PTEN in CSCC have been less studied.

miR-141, as a member of miR-200 family, is located at 12p13.3 and plays an important role in regulating tumor cell proliferation, migration, differentiation, and apoptosis [13]. Studies have shown that miR-141 is abnormally expressed in colorectal cancer, non-small-cell lung cancer, and gastric cancer and is increased in ovarian cancer, breast cancer, prostate cancer, renal cell cancer, and bladder cancer [14,15]. miR-340 is located at 5q35.3 and regulates the cell cycle by regulating various target proteins, which in turn affects tumor invasion and metastasis [16,17,18]. It functions as a tumor suppressor and is downregulated in solid tumors such as breast cancer, prostate cancer, gastric cancer, osteosarcoma, and melanoma [19,20]. In this study, we found that miR-141 expression was mostly upregulated in CSCC, and it showed a downward trend in tissues from CSCC to HSIL and normal squamous epithelium. In contrast, miR-340 expression was mostly downregulated in CSCC and had an upward trend in the tissues from CSCC to HSIL and normal squamous epithelium. These results indicate that miR-141 and miR-340 may play important regulatory roles in the development of normal cervical squamous epithelium into HSIL and even CSCC.

We found that miR-141 was closely related to the gross type, differentiation, uterine corpus invasion, nerve invasion, vagina invasion, and FIGO stage in CSCC. At the same time, miR-340 was closely related to the tumor size, differentiation, nerve invasion, lymph node metastasis, and FIGO stage in CSCC. These results indicate that miR-141 and miR-340 are involved in the progression of CSCC and can be used as indicators for the progress evaluation of CSCC, but the specific mechanism is yet to be further studied.

In addition, we also found that miR-141 and miR-340 showed a negative correlation in CSCC and HSIL. But the difference was not statistically significant. These results suggest that miR-141 and miR-340 may have opposite regulatory functions in the process from HSIL to CSCC. However, this conclusion still needs to be verified by further expanding the sample size. The loss expression of PTEN, the common target gene of miR-141 and miR-340, is the molecular mechanism involved in various malignant tumors [21,22,23,24]. Our results showed that the PTEN-positive expression rate in CSCC was 42.3%. In addition, PTEN expression may be related to age, but not to the ethnic group, gross type, tumor size, differentiation, invasion depth, uterine corpus invasion, vascular invasion, nerve invasion, vagina invasion, lymph node metastasis, and FIGO stage. Therefore, we speculate that miR-141 and miR-340 might have not only common but also different regulatory effects on the pathogenesis from normal cervical squamous epithelium to HSILs and invasive CSCC.

## 5 Conclusion

In conclusion, miR-141 and miR-340 participated in the process of abnormal hyperplasia of cervical epithelium to HSIL and in the development of CSCC. However, the underlying mechanisms may be different. We found that miR-141 and miR-340 had higher sensitivity and specificity for the diagnosis of CSCC and the diagnosis of HSIL, respectively, suggesting that the combination of miR-141 and miR-340 could be used for the diagnosis of CSCC and HSIL. However, the regulatory mechanism of miR-141 and miR-340 on target gene PTEN still needs further research and verification in cell models or animal models. Moreover, our research has good clinical application prospects. In clinical practice, the detection of miR-141 and miR-340, as well as the immunohistochemical detection of PTEN, can be applied to the early diagnosis of cervical intraepithelial lesions and CSCC, which would provide a basis for diagnosis, treatment, and prognosis of CSCC and squamous intraepithelial lesions.
